# Ultrathin Two‐Dimensional Plasmonic PtAg Nanosheets for Broadband Phototheranostics in Both NIR‐I and NIR‐II Biowindows

**DOI:** 10.1002/advs.202100386

**Published:** 2021-07-11

**Authors:** Ying Zhang, Qi Shen, Qi Li, Panpan He, Jinyan Li, Feng Huang, Jing Wang, Yefan Duan, Chuang Shen, Faisal Saleem, Zhimin Luo, Lianhui Wang

**Affiliations:** ^1^ State Key Laboratory for Organic Electronics and Information Displays and Jiangsu Key Laboratory for Biosensors Institute of Advanced Materials (IAM) Jiangsu National Synergetic Innovation Center for Advanced Materials (SICAM) College of Electronic and Optical Engineering and College of Microelectronic Nanjing University of Posts and Telecommunications 9 Wenyuan Road Nanjing 210023 China; ^2^ Department of Human Anatomy, School of Basic Medical Sciences Key Laboratory of Brain Aging and Neurodegenerative Diseases of Fujian Province Fujian Medical University 1 Xueyuan Road Fuzhou 350122 China; ^3^ Key Laboratory of Flexible Electronics (KLOFE) & Institute of Advanced Materials (IAM) Nanjing Tech University (NanjingTech) 30 South Puzhu Road Nanjing 211816 China

**Keywords:** broadband near‐infrared biowindow, photoacoustic imaging, phototheranostics, photothermal therapy, PtAg nanosheets

## Abstract

Broadband near‐infrared (NIR) photothermal and photoacoustic agents covering from the first NIR (NIR‐I) to the second NIR (NIR‐II) biowindow are of great significance for imaging and therapy of cancers. In this work, ultrathin two‐dimensional plasmonic PtAg nanosheets are discovered with strong broadband light absorption from NIR‐I to NIR‐II biowindow, which exhibit outstanding photothermal and photoacoustic effects under both 785 and 1064 nm lasers. Photothermal conversion efficiencies (PCEs) of PtAg nanosheets reach 19.2% under 785 nm laser and 45.7% under 1064 nm laser. The PCE under 1064 nm laser is higher than those of most reported inorganic NIR‐II photothermal nanoagents. After functionalization with folic acid modified thiol‐poly(ethylene glycol) (SH‐PEG‐FA), PtAg nanosheets endowed with good biocompatibility and 4T1 tumor‐targeted function give high performances for photoacoustic imaging (PAI) and photothermal therapy (PTT) in vivo under both 785 and 1064 nm lasers. The effective ablation of tumors in mice can be realized without side effects and tumor metastasis by PAI‐guided PTT of PtAg nanosheets under 785 or 1064 nm laser. The results demonstrate that the prepared PtAg nanosheets with ultrathin thickness and small size can serve as a promising phototheranostic nanoplatform for PAI‐guided PTT of tumors in both NIR‐I and NIR‐II biowindows.

## Graphical abstract

Plasmonic ultrathin PtAg nanosheets are discovered to have strong broadband photothermal and photoacoustic effect for photothermal therapy (PTT) and photoacoustic imaging (PAI) of tumors in both the first near‐infrared (NIR‐I) and second near‐infrared (NIR‐II) biowindows.

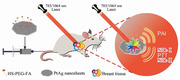

## 1 Introduction

Photothermal therapy (PTT) and photoacoustic imaging (PAI) of tumors have been intensively explored in recent years.^[^ ^1^ ^]^ PTT based on near‐infrared (NIR) light gives a noninvasive avenue for the ablation of tumors through local hyperthermia induced by photothermal agents.^[^ ^2^ ^]^ PAI under NIR light can help in deeply visualizing the tumor location within a tissue to provide structural and functional information for preclinical studies.^[^ ^3^ ^]^ Until now, many materials have been investigated as photothermal and photoacoustic agents, such as organic molecules,^[^ ^4^ ^]^ polymers,^[^ ^5^ ^]^ carbon nanomaterials,^[^ ^6^, ^7^, ^8^ ^]^ transition metal dichalcogenides,^[^ ^9^, ^10^ ^]^ noble metal nanostructures,^[^ ^11^ ^]^ and so on. Noble metal nanostructures have attracted extensive attention as a kind of photothermal and photoacoustic agents due to the strong light absorption derived from their surface plasmonic effects. In the development of noble metal nanomaterials for PTT and PAI, some noble metal nanostructures are found to have photothermal and photoacoustic characteristics for PTT and PAI, such as Au nanorods,^[^ ^12^ ^]^ Au nanovesicles,^[^ ^13^ ^]^ Ag nanoplates,^[^ ^14^ ^]^ Pd nanoparticles,^[^ ^15^ ^]^ Pt nanoparticles,^[^ ^16^ ^]^ etc., but their light absorption and related researches mainly lie in the first NIR (NIR‐I) biowindow (650–950 nm). Although regulating the shape and size of noble metal nanostructures has been devoted to tuning their surface plasmonic resonance (SPR) wavelength to the second NIR (NIR‐II) biowindow (1000‐1700 nm), their sizes are often too large to meet the requirement of biological applications.^[^ ^11^, ^14^, ^17^, ^18^, ^19^ ^]^

Nowadays, since NIR‐II laser exhibits intrinsic advantages of deeper tissue penetration and higher maximum permissible exposure (MPE), great interest is growing to prepare unique nanomaterials with the light absorption extended from NIR‐I to NIR‐II biowindow.^[^ ^20^, ^21^, ^22^, ^23^ ^]^ This kind of nanomaterials has both NIR‐I and NIR‐II photothermal and photoacoustic properties, which are meaningful for the directly comparative study of their PTT and PAI in the NIR‐I and NIR‐II regions.^[^ ^20^, ^21^, ^22^ ^]^ However, few noble metal nanostructures have been reported on their photothermal and photoacoustic characteristics in both NIR‐I and NIR‐II biowindows. Pt nanostructures as a kind of traditionally catalytic nanomaterials have the potential to extend their SPR wavelengths to NIR‐II biowindow for preparing broadband photothermal and photoacoustic agents due to their high‐order longitudinal SPR mode locating in the NIR zone. To the date, there has been almost no study of Pt nanostructures especially two‐dimensional (2D) Pt‐based nanostructures for PTT and PAI of tumors in both NIR‐I and NIR‐II biowindows.

**Scheme 1:**
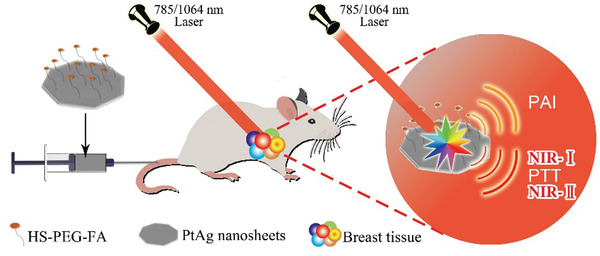
Ultrathin PtAg nanosheets functionalized with SH‐PEG‐FA for 4T1 tumor‐targeted photothermal therapy (PTT) guided by photoacoustic imaging (PAI) under both 785 and 1064 nm lasers.

We herein synthesize 2D PtAg nanosheets by a simple two‐step wet‐chemistry method, which are discovered to have broadband light absorption from NIR‐I to NIR‐II biowindow (**Scheme** **1**). This kind of plasmonic PtAg nanosheets differs from traditional Au nanorods and other noble metal nanoparticles, demonstrating stronger photothermal and photoacoustic effects than reported Ag nanoplates^[^ ^14^ ^]^ and Pt nanoparticles^[^ ^16^ ^]^ in NIR‐II biowindow. Their small size less than 50 nm and ultrathin thickness further ensure them to be used as a novel phototherapeutic agent for PTT and PAI in vivo. PtAg nanosheets functionalized with folic acid modified thoil‐poly(ethylene glycol) (SH‐PEG‐FA) can realize targeted ablation of 4T1 tumors through PAI guided PTT under both NIR‐I and NIR‐II lasers.

## 2 Results and Discussion

**Figure 1:**
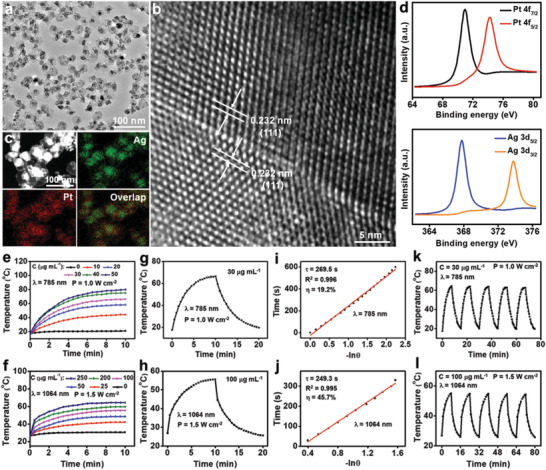
(a) Transmission electron microscopy (TEM), (b) high‐resolution TEM (HRTEM), and (c) corresponding energy dispersive X‐ray spectroscopy (EDS) mapping images of PtAg nanosheets. (d) High‐resolution Pt 4f and Ag 3d X‐ray photoelectron spectroscopy (XPS) spectra of PtAg nanosheets. Photothermal heating curves of PtAg nanosheets aqueous suspensions with different concentrations under the irradiation of (e) 785 nm and (f) 1064 nm lasers. Heating of PtAg nanosheets aqueous suspensions with (g) 785 nm and (h) 1064 nm lasers irradiation for 10 min and then cooling at ambient environment. Thermal equilibrium time constant of PtAg nanosheets aqueous suspensions under (i) 785 nm and (j) 1064 nm lasers determined by fitting the time data versus negative natural logarithm of the driving force temperature from the cooling period. Photothermal stability of PtAg nanosheets aqueous suspensions under (k) 785 nm and (l) 1064 nm lasers through repeated heating and cooling measurements. The power density of 785 and 1064 nm lasers used in the experiments are 1.0 and 1.5 W cm^−2^, respectively.

The prepared PtAg nanosheets are characterized by X‐ray diffraction (XRD), transmission electron microscopy (TEM) and high‐resolution TEM (HRTEM), energy dispersive X‐ray spectroscopy (EDS) elemental mapping images, and X‐ray photoelectron spectroscopy (XPS). XRD pattern in Figure S1 (Supporting Information) gives the peaks at 2*θ* of 38.8°, 44.9°, 65.7°, and 78.5°, corresponding to (111), (200), (220), and (311) crystal plane of PtAg nanostructure.^[^ ^24^ ^]^ As observed from **Figure** **1**a, the obtained PtAg nanosheets show the ultrathin 2D shape with an average lateral size of 37.6 nm. Thickness analysis from HRTEM image of an erective PtAg nanosheet (Figure S2, Supporting Information) gives the value of ≈1.5 nm, indicating its ultrathin nanostructure. HRTEM image in Figure 1b displays the crystalline fringes with the interplanar spacings of 0.232 nm corresponding to (111) plane of face‐centered‐cubic PtAg alloyed structures.^[^ ^24^ ^]^ The EDS elemental mapping images of Pt and Ag elements are clearly observed from Figure 1c. The high‐resolution Pt 4f XPS spectrum (Figure 1d) gives the binding energies at 71.0 and 74.3 eV, which are attributed to Pt 4f_7/2_ and Pt 4f_5/2_, respectively.^[^ ^25^ ^]^ The XPS peaks at 367.8 and 373.8 eV (Figure 1d) correspond to Ag 3d_5/2_ and Ag 3d_3/2,_ respectively.^[^ ^25^ ^]^ These XPS characterizations further confirm the structure and composition of prepared PtAg nanosheets.

Optical absorption and photothermal property of PtAg nanosheets are investigated. As shown in Figure S3a (Supporting Information), PtAg nanosheets show broadband absorbance from 400 to 1200 nm. The absorbance of PtAg nanosheets increases with their concentrations. The extinction coefficients (*ε*) of PtAg nanosheets at the wavelength of 785 (Figure S3b, Supporting Information) and 1064 nm (Figure S3c, Supporting Information) are estimated to be 27.2 and 3.1 L g^−1^ cm^−1^ according to the Beer–Lambert law, respectively. The temperatures of PtAg nanosheet aqueous suspensions rise with the power density of laser (Figure S4, Supporting Information) and their concentrations (Figure 1e,f). After the irradiation of 785 nm laser for 6 min, the temperature of PtAg nanosheet aqueous suspension (30 µg mL^−1^) increases fastly from 17 °C to 62 °C (Figure 1g). Furthermore, the temperature of PtAg nanosheet aqueous suspensions (100 µg mL^−1^) can also increase by 28 °C under the irradiation of 1064 nm laser (1.5 W cm^−2^) for 6 min (Figure 1h). The corresponding photothermal images of PtAg nanosheets with different concentrations are presented in Figure S5 (Supporting Information). The photothermal conversion efficiencies (PCEs, *η*) of PtAg nanosheets are calculated based on the results of time constant for heat transfer and the maximum steady‐state temperature, which give the values of 19.2% under 785 nm laser and 45.7% under 1064 nm laser (Figure 1i,j). The comparison of PCE of PtAg nanosheets with other photothermal nanoagents under 1064 nm laser can be seen in Table S1 (Supporting Information). The PCE of PtAg nanosheets under 1064 nm laser (45.7%) is higher than those of previously reported Pt nanoparticles with different sizes (22.98–30.88%),^[^ ^16^ ^]^ worm‐like Pt nanoparticles (38.9%),^[^ ^26^ ^]^ Pt nanocubes (32.3%),^[^ ^27^ ^]^ porous Pt superstructures (43.2%),^[^ ^27^ ^]^ Au@Cu_2‐x_S (43.5%),^[^ ^28^ ^]^ Au nanoplates@TiO_2_ (42.05%),^[^ ^19^ ^]^ Au‐Cu_9_S_5_ nanoparticles (37.0%),^[^ ^29^ ^]^ Au/Ag alloy double nanoshell (28.3%),^[^ ^30^ ^]^ Cu_3_BiS_3_ nanorods (40.7%),^[^ ^31^ ^]^ Bi@C nanoparticles (42.32%),^[^ ^32^ ^]^ Fe_3_O_4_@CuS (19.2%),^[^ ^33^ ^]^ MoO_x_ nanoparticles (37.4%),^[^ ^34^ ^]^ and 1T‐MoS_2_ nanodots (43.3%).^[^ ^35^ ^]^ Photostability measurements show that there is no variation for heating/cooling curves after 5‐cycle repeated heating and cooling measurements (Figure 1k,l), indicating that PtAg nanosheets can be a kind of durable photothermal agent for cancer treatments.

**Figure 2:**
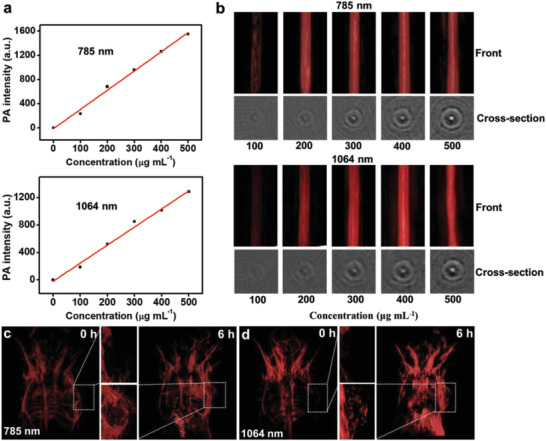
(a) Photoacoustic signals of deionized water and PtAg nanosheet aqueous suspensions (100, 200, 300, 400, 500 µg mL^−1^) under 785 and 1064 nm lasers. (b) Photoacoustic imaging (PAI) of PtAg nanosheet aqueous suspensions (100, 200, 300, 400, 500 µg mL^−1^) in the front and cross‐section of photoacoustic tubes under 785 and 1064 nm lasers. In vivo PAI of the 4T1‐bearing mouse body and tumor site under (c) 785 nm and (d) 1064 nm lasers after intravenous injection of SH‐PEG‐FA functionalized PtAg nanosheets.

Owing to the strong absorbance and marked PCE of PtAg nanosheets in the NIR region, their photoacoustic performance in vitro is further explored. Ti:Saph (785 nm, ≈160 mJ pulse^−1^) and Nd:YAG (1064 nm, ≈300 mJ pulse^−1^) lasers are used for measuring the photoacoustic signals of PtAg nanosheets. The relatively large output energy of lasers is used to compensate for the energy losses caused by the optical absorption of water layer between the laser fiber bundle and samples. As shown in **Figure** **2**a, photoacoustic signals of PtAg nanosheets display a great linear correlation relationship with their concentrations under both 785 and 1064 nm lasers, which is advantageous for quantitatively photoacoustic analysis. Photoacoustic images of PtAg nanosheet aqueous suspensions with various concentrations (100, 200, 300, 400, 500 µg mL^−1^) in the front and cross‐section of photoacoustic tubes under 785 or 1064 nm laser can be observed in the Figure 2b. The photoacoustic intensity of PtAg nanosheet aqueous suspensions is significantly higher than that of deionized water, and increases notably with their concentrations under 785 and 1064 nm lasers. These experimental results demonstrate that PtAg nanosheets have excellent photoacoustic performance and are promising for PAI in both NIR‐I and NIR‐II biowindows.

Considering the possible biological applications of PtAg nanosheets, biocompatible SH‐PEG‐FA with the function of targeting 4T1 breast cancer tumor is used to modify PtAg nanosheets. As shown in Figure S6 (Supporting Information), the morphology of PtAg nanosheets is almost not changed after chemical functionalization with SH‐PEG‐FA. Zeta potential measurements (Figure S7, Supporting Information) show the transformation of zeta potential from 7.9 mV for PtAg nanosheets to −11.8 mV for SH‐PEG‐FA functionalized PtAg nanosheets in phosphate buffered saline (PBS) (pH = 7.4, 10 × 10^−3^ m), indicating their improvement of colloidal stability after functionalization of PtAg nanosheets with SH‐PEG‐FA. The chemical stability of SH‐PEG‐FA functionalized PtAg nanosheets is studied. As shown in Figure S8 (Supporting Information), there is no aggregation or change of absorbance at 1064 nm for SH‐PEG‐FA functionalized PtAg nanosheets kept in the deionized water, PBS (pH = 7.4, 10 × 10^−3^ m), physiological buffered saline (PBS + 0.9%NaCl) or Roswell Park Memorial Institute‐1640 (RPMI‐1640) medium for 4 d. We also evaluate their cytotoxicity by a traditional tetrazolium‐based colorimetric assay (MTT) method using human cervix cancer (HeLa) and 4T1 cells as cell models. As shown in Figures S9a and S10a (Supporting Information), the cell viabilities are still more than 90% after HeLa and 4T1 cells are incubated with SH‐PEG‐FA functionalized PtAg nanosheets (75 µg mL^−1^) for 24 h, indicating the good biocompatibility of SH‐PEG‐FA functionalized PtAg nanosheets. However, the cell viability at the same concentration of SH‐PEG‐FA functionalized PtAg nanosheets significantly decreases after the irradiation of 785 or 1064 nm laser for 10 min (Figures S9a and S10a, Supporting Information), suggesting their remarkable photothermal killing effect. Fluorescent confocal imaging of HeLa and 4T1 cells stained with propidium iodide (PI) is carried out after HeLa and 4T1 cells are incubated with or without SH‐PEG‐FA functionalized PtAg nanosheets for 12 h and then irradiated with 785 or 1064 nm laser for 10 min. As shown in Figures S9b and S10b (Supporting Information), most of the HeLa and 4T1 cells were killed after being incubated with SH‐PEG‐FA functionalized PtAg nanosheets and then irradiated by 785 or 1064 nm laser. The cellular experiments demonstrate that SH‐PEG‐FA functionalized PtAg nanosheets are potential for biological applications as an effective PTT agent.

Encouraged by the excellent photothermal and photoacoustic properties of SH‐PEG‐FA functionalized PtAg nanosheets in vitro, we further investigate the application of SH‐PEG‐FA functionalized PtAg nanosheets for PAI‐guided PTT in vivo. As shown in Figure 2c,d and Figure S11 (Supporting Information), the remarkable enhancement of photoacoustic signals in tumor sites can be clearly observed under 785 and 1064 nm lasers after 4T1 tumor‐bearing mice are intravenously injected with 200 µL of SH‐PEG‐FA functionalized PtAg nanosheets suspension (500 µg mL^−1^) for 6 h. It is suggested that SH‐PEG‐FA functionalized PtAg nanosheets have strong photoacoustic performance with good permeability and retention effect in tumor sites for PAI of 4T1 tumor. The laser fluences measured at the skin of mice are ≈0.92 mJ pulse^−1^ cm^2^ for 785 nm laser and ≈1.2 mJ pulse^−1^ cm^2^ for 1064 nm laser, which meet the requirement of international ANSI Standard (<20 mJ pulse^−1^ cm^2^).

**Figure 3:**
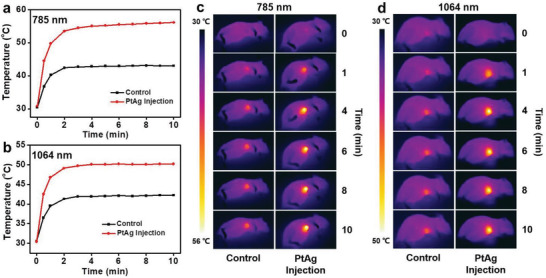
Photothermal heating curves of 4T1 tumors under (a) 785 and (b) 1064 nm lasers after intravenous injection of SH‐PEG‐FA functionalized PtAg nanosheets for 6 h. Photothermal imaging of 4T1 tumors under (c) 785 and (d) 1064 nm lasers after intravenous injection of PtAg nanosheets for 6 h.

PTT of SH‐PEG‐FA functionalized PtAg nanosheets in vivo is studied under the guidance of PAI. Photothermal heating curves of 4T1 tumors in **Figure** **3**a,b show that the temperatures at the 4T1 tumor sites rapidly reach 56.1 and 50.2 °C under the irradiation of 785 and 1064 nm lasers for 2 min, respectively, while the skin temperature of mice without injection of PtAg nanosheets can only reach 43.0 °C and 42.2 °C. It arises from the enrichment effect of SH‐PEG‐FA functionalized PtAg nanosheets at 4T1 tumor sites and their prominent photothermal properties in both NIR‐I and NIR‐II biowindows. Photothermal imaging of 4T1 tumors after intravenous injection of SH‐PEG‐FA functionalized PtAg nanosheets for 6 h under 785 and 1064 nm lasers (Figure 3c,d) further proves it. Hematoxylin and eosin (H&E) staining of 4T1 tumor slices of mice with different treatments (Figure S12, Supporting Information) displays that 4T1 tumor tissues are mostly damaged by the photothermal effect of SH‐PEG‐FA functionalized PtAg nanosheets under 785 or 1064 nm laser, while the tumor slices of mice treated by only SH‐PEG‐FA functionalized PtAg nanosheets without NIR lasers show no significant tumor tissue necrosis. Hyperthermia caused by enriched SH‐PEG‐FA functionalized PtAg nanosheets at 4T1 tumor sites results in unfolding of proteins in short time and leads to irreversible damages to cancer cells, while momentary increasing of the skin temperature to 42–43 °C in the irradiation areas does not destroy the normal tissues. Immunohistochemical staining of proliferating cellular nuclear antigen (PCNA) in tumor slice was also performed to assess the effect of SH‐PEG‐FA functionalized PtAg nanosheets for PTT. It can be observed from Figure S13a (Supporting Information) that abundant PCNA‐positive cells are present in the control group. By contrast, there is almost no PCNA‐positive cell in the experimental groups injected with SH‐PEG‐FA functionalized PtAg nanosheets and irradiated with 785 or 1064 nm laser (Figure S13b,c, Supporting Information). These results of PCNA assay are consistent with those of H&E staining of tumor tissue, further suggesting the strong photothermal effect of SH‐PEG‐FA functionalized PtAg nanosheets to inhibit tumor proliferation.

**Figure 4:**
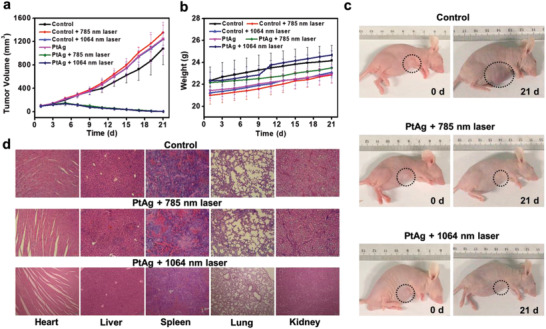
(a) Relative tumor volumes and (b) body weights of the mice after various treatments for different time. (c) Photographs of the mice before and after various treatments for 21 d. (d) Hematoxylin and eosin (H&E) stained images of major organs (heart, liver, spleen, lung, and kidney) from the mice groups incubated for 21 d after no treatment (control) or treatment with SH‐PEG‐FA functionalized PtAg nanosheets and 785 nm laser (PtAg + 785 nm laser) or 1064 nm laser (PtAg + 1064 nm laser).

The tumor volumes and weights of mice after various treatments are recorded. As shown in **Figure** **4**a, the tumor volumes for the groups of mice without treatments by SH‐PEG‐FA functionalized PtAg nanosheets or NIR lasers always increase in the period of 21 d, while the tumor volumes of mice treated by SH‐PEG‐FA functionalized PtAg nanosheets and NIR lasers increase slightly in the beginning 4.5 d and then gradually shrink in the later 16.5 d. The tumors are almost completely eliminated after treatment for 21 d. The body weights of mice in all groups show no obvious variation (Figure 4b), indicating a good biosafety and high therapeutic efficacy of PTT using SH‐PEG‐FA functionalized PtAg nanosheets as a PTT agent. Photographs of the mice before and after different treatments (Figure 4c) present the evident photothermal therapeutic effect of SH‐PEG‐FA functionalized PtAg nanosheets under the irradiation of 785 or 1064 nm laser. H&E staining images of major organs from the mice treated by SH‐PEG‐FA functionalized PtAg nanosheets and 785 or 1064 nm laser (Figure 4d) display that there are no degeneration and necrosis of myocardial cells in the heart. No degeneration or necrosis of liver cells is observed in the lung. The normal megakaryocytes are visible in the spleen and there is no significant change for the number of acini lienalis in the white pulp. The lung is clearly structured and there is no exudate in the alveolar cavity at all levels. The balloon cavity is clear and has no necrosis. There is no atrophy or enlargement of the glomerulus in the kidney. Analytical results for the blood routines in Table S2 (Supporting Information) show no statistically differences among the mice after PTT and in control groups, suggesting the good safety of SH‐PEG‐FA functionalized PtAg nanosheets for PTT in vivo. All these results confirm that SH‐PEG‐FA functionalized PtAg nanosheets have good biocompatibility in vivo for PTT of 4T1 tumors under both NIR‐I and NIR‐II lasers without side effects and tumor metastasis.

## 3 Conclusion

In summary, ultrathin 2D PtAg nanosheets are found to have good photothermal and photoacoustic properties in both NIR‐I and NIR‐II biowindows. The unique 2D noble metal nanosheets exhibit high PCEs (19.2% at 785 nm laser and 45.7% at 1064 nm laser). Photoacoustic signals of PtAg nanosheets are strong in the physiological solution and proportional to their concentrations. Functionalization of PtAg nanosheets by SH‐PEG‐FA endows them with good biocompatibility and 4T1 tumor‐targeted ability in vivo. Experimental results show that 4T1 tumors in mice can be efficiently ablated by SH‐PEG‐FA functionalized PtAg nanosheets through PAI‐guided PTT under 785 or 1064 nm laser without side effects and tumor metastasis. It is indicated that the plasmonic ultrathin PtAg nanosheets are promising to be a novel photothermal and photoacoustic agent for highly efficient phototherapeutics of cancers in both NIR‐I and NIR‐II biowindows.

## Conflict of Interest

The authors declare no conflict of interest.

## Supporting information

Supporting Information

## Data Availability

Research data are not shared.
