# Supplementary material for: Ultrathin Two‐Dimensional Plasmonic PtAg Nanosheets for Broadband Phototheranostics in Both NIR‐I and NIR‐II Biowindows
Source: Adv Sci (Weinh). 2021 Jul 11;8(17):2100386. doi: 10.1002/advs.202100386 (PMC8425935; doi:10.1002/advs.202100386)
Supplement: Supplementary file 1 — Supporting Information [file ADVS-8-2100386-s001.pdf]

© 2021 Wiley-VCH GmbH

ADVANCED SCIENCE
Open Access

Supporting Information

for *Adv. Sci.,* DOI: 10.1002/advs.202100386

Ultrathin Two-Dimensional Plasmonic PtAg Nanosheets for Broadband Phototheranostics in both NIR-I and NIR-II Biowindows

Ying Zhang, Qi Shen, Qi Li, Panpan He, Jinyan Li, Feng Huang, Jing Wang, Yefan Duan, Chuang Shen, Faisal Saleem,* Zhimin Luo,* Lianhui Wang**

Ultrathin Two-Dimensional Plasmonic PtAg Nanosheets for Broadband Phototheranostics in both NIR-I and NIR-II Biowindows

Ying Zhang, Qi Shen, Qi Li, Panpan He, Jinyan Li, Feng Huang, Jing Wang, Yefan Duan, Chuang Shen, Faisal Saleem,* Zhimin Luo,* Lianhui Wang**

Dr. Y. Zhang, Q. Shen, Q. Li, P. He, J. Li, J. Wang, Y. Duan, C. Shen, Prof. Z. Luo, Prof. L. Wang

State Key Laboratory for Organic Electronics and Information Displays & Jiangsu Key Laboratory for Biosensors, Institute of Advanced Materials (IAM), Jiangsu National Synergetic Innovation Center for Advanced Materials (SICAM), College of Electronic and Optical Engineering & College of Microelectronic, Nanjing University of Posts and Telecommunications, 9 Wenyuan Road, Nanjing 210023, China.

Email: iamzmluo@njupt.edu.cn, iamlhwang@njupt.edu.cn.

Prof. F. Saleem

Key Laboratory of Flexible Electronics (KLOFE) & Institute of Advanced Materials (IAM), Nanjing Tech University (NanjingTech), 30 South Puzhu Road, Nanjing 211816, China.

Email: iamfaisalsaleem@njtech.edu.cn

Dr. F. Huang

Department of Human Anatomy, School of Basic Medical Sciences, Key Laboratory of Brain Aging and Neurodegenerative Diseases of Fujian Province, Fujian Medical University, 1 Xueyuan Road, Fuzhou 350122, China.

Email: huangfeng@fjmu.edu.cn

Experimental section

Materials. Silver nitrate (99%), platinum (II) acetylacetonate (99.98%), potassium iodide (99.99%), poly(vinylpyrrolidone) (PVP) (average molecular weight = 10 K), tris(hydroxymethyl) aminomethane (99.8%), folic acid modified thoil-poly(ethylene glycol) (SH-PEG-FA) (>95%), propidium iodide (PI), formaldehyde solution (37 wt%), 3-(4,5)-dimethylthiazol-2-yl)-2,5-diphenyl tetrazolium bromide (MTT) and formamide (99.5%) were all purchased from Sigma-Aldrich (Nanjing, China) and used without further purification. Acetone (99.5%) and ethanol (99.7%) were purchased from Wuxi City Yasheng Chemical Co., Ltd (Wuxi, China).

Synthesis of PtAg nanosheets. PtAg nanosheets were prepared by using a modified procedure based on a previous report.[1] In a typical synthesis, a homogenous solution is prepared by mixing PVP (400 mg) and tris(hydroxymethyl) aminomethane (100 mg) in formaldehyde solution (4 mL). Then this homogenous solution is heated at 190 ^{o}C for 3 h in a 23 mL Teflon-lined stainless steel autoclave. After cooling the autoclave, acetone (30 mL) was added and the suspension was centrifuged at 10000 rpm for 5 min. A dark-brown gel-like materials was obtained, which was washed with acetone for three times and dried at 115 ^{o}C for 5 min. A homogeneous solution containing silver nitrate (4.3 mg), platinum (II) acetylacetonate (7.8 mg), potassium iodide (120 mg) and formamide solvent (4 mL) were then mixed with the aforementioned dark-brown gel and heated at 130 ^{o}C for 3 h in a 23 mL Teflon-lined stainless steel autoclave. Finally, the resultant PtAg nanosheets were separated by using a mixture of acetone (10 mL) and ethanol (2 mL), followed by centrifugation for two times at 10000 rpm for 5 min.

Functionalization of PtAg nanosheets with SH-PEG-FA. 10 mL of PtAg nanosheets aqueous suspension (0.5 mg mL^{-1}) was mixed with 50 mg of SH-PEG-FA. After ultrasonication for 4 h and stirring for 8 h, excessive SH-PEG-FA was removed by centrifugation at 25000 rpm for 15 min and the product was repeatedly rinsed with deionzed water. The resultant SH-PEG-FA functionalized PtAg nanosheets were redispersed in the deionized water for next use.

Characterizations. The morphologies and structures of PtAg nanosheets were characterized by transmission electron microscopy (TEM) and high-resolution TEM (HRTEM) (JEOL JEM-2100F) at an accelerating voltage of 200 kV. X-ray photoelectron spectroscopy (XPS) was performed on a PHI 5000 VersaProbe with Al Kα (hν = 1486.6 eV) as the excitation source. Raman spectra were recorded on a micro-Raman spectroscopy system (Renishaw, UK) equipped with a 532 nm laser. Photothermal effect and imaging were measured by a thermal infrared imager (Fotric 225RD). The power density of laser irradiation was measured by a digital power meter (PM100D, Thorlabs, USA). The UV-vis-NIR absorption spectra were recorded on a UV-vis-NIR spectrophotometer (UV 3600, Shimadzu, Japan). The concentration of PtAg nanosheets was determined by Inductively Coupled Plasma Optical Emission Spectrometer (ICP-OES, Optima 5300DV, Perkin Elmer). The photoacoustic signal was measured by LOIS-3D (TomoWave Laboratories, USA).

Photothermal properties of PtAg nanosheets under 785 and 1064 nm lasers. 250 μL of PtAg nanosheets aqueous suspensions with various concentrations (10, 20, 30, 40 and 50 μg mL^{-1}) was respectively loaded in the 250 μL centrifuge tubes and irradiated by 785 nm laser at the power density of 1.0 W cm^{-2} for 10 min. To measure the photothermal performance of PtAg nanosheets under 1064 nm laser, 250 μL of PtAg nanosheets aqueous suspensions (25, 50, 100, 200 and 250 μg mL^{-1}) were respectively placed in the 250 μL centrifuge tubes and irradiated by 1064 nm laser (1.5 W cm^{-2}) for 10 min. The digital photothermal imaging system was used to visually monitor the temperature changes of PtAg nanosheets aqueous suspensions. Heating of PtAg nanosheets aqueous suspension for 8 min and then naturely cooling it through five repeated lasers on/off cycles were carried out to evaluate its photothraman stability. The concentrations of PtAg nanosheets aqueous suspensions used to measure photothermal stability are 30 μg mL^{-1} for 785 nm laser (1.0 W cm^{-2}) and 100 μg mL^{-1} for 1064 nm laser (1.5 W cm^{-2}). The determination of photothermal conversion efficiency (η) was referring to the previous reports.[2, 3]

Cytotoxicity and photothermal killing effect of SH-PEG-FA functionalized PtAg nanosheets in vitro. HeLa cells were seeded in 96-well plates with a density of 10^4 cells per well and incubated in Dulbecco's modified Eagle's medium (DMEM) containing 10% fetal bovine serum (FBS) and 1% penicillin/ streptomycin at 37 °C under 5 % CO_2 for 24 h. SH-PEG-FA functionalized PtAg nanosheets with various concentrations from 0 to 150 μg mL^{-1} were added and then incubated for 24 h. Each well was irradiated with 785 nm laser (1.0 W cm^{-2}) or 1064 nm laser (1.5 W cm^{-2}) for 10 min. The cell viability was assayed by adding 20 μL of MTT PBS solution (10 mM, pH = 7.4) (5 mg mL^{-1}) to each well and the cells were incubated with MTT at 37 °C for 4 h. After that, the MTT solution was removed from all samples and 200 μL of DMSO was added to dissolve the eventually formed formazan crystals. The absorbance at the wavelength of 490 nm was measured by microplate reader to confirm the cell viability. The following formula was used to calculate the inhibition of cell growth: Cell viability (%) = (mean absorbance value of treatment group/mean absorbance value of control) × 100%.

Confocal imaging after PI staining was further carried out to visually present the photothermal cytotoxicity of PtAg nanosheets. Five groups of HeLa cells were incubated and treated with different conditions as follows: (1) incubation with PBS (10 mM, pH = 7.4) for 8 h (control group); (2) incubation with SH-PEG-FA functionalized PtAg nanosheets aqueous suspension (30 μg mL^{-1}) for 8 h; (3) incubation with SH-PEG-FA functionalized PtAg nanosheets aqueous suspension (30 μg mL^{-1}) for 8 h and then exposion to 785 nm laser (1.0 W cm^{-2}) for 10 min; (4) incubation with SH-PEG-FA functionalized PtAg nanosheets aqueous suspension (150 μg mL^{-1}) for 8 h; (5) incubation with SH-PEG-FA functionalized PtAg nanosheets aqueous suspension (150 μg mL^{-1}) for 8 h and then exposion to 1064 nm laser (1.5 W cm^{-2}) for 10 min. HeLa cells were washed twice with PBS (10 mM, pH = 7.4) and then stained with PI (5 μM) for 20 min to observe the state of HeLa cells through a confocal microscope.

Cytotoxicity and photothermal killing effect of SH-PEG-FA functionalized PtAg nanosheets towards 4T1 cells were carried out according to the similar operations except the incubation conditions of 4T1

cells. 4T1 cells were incubated in Roswell Park Memorial Institute medium (RPMI-1640) containing 10% FBS and 1% penicillin/streptomycin at 37 °C under 5% CO_2.

Photoacoustic measurements of SH-PEG-FA functionalized PtAg nanosheets in vitro and in vivo. SH-PEG-FA functionalized PtAg nanosheets aqueous suspensions (100, 200, 300, 400 and 500 μg mL^{-1}) were injected into different photoacoustic glass tubes and then the photoacoustic tubes were put into the LOIS-3D machine to collect the photoacoustic intensity under 785 or 1064 nm laser. To measure the photoacoustic signals in vivo, the 4T1 tumor-bearing mice were intravenously injected with 200 μL of 500 μg mL^{-1} SH-PEG-FA functionalized PtAg nanosheets suspension (0.9% NaCl aqueous solution) and then the mice were put into the LOIS-3D machine to collect the photoacoustic signals of tumor under 785 or 1064 nm laser.

In vivo photothermal therapy of SH-PEG-FA functionalized PtAg nanosheets. Nude mice (6 weeks old, weight 20-25 g) were used for experiments in vivo. The tumor models were established by subcutaneous injection of 4T1 cells in the right axilla area of each mouse. When the tumor volumes reach around 100 mm^3, the mice were intravenously injected with 200 μL of 500 μg mL^{-1} SH-PEG-FA functionalized PtAg nanosheets suspension (0.9% NaCl aqueous solution). After incubation for 6 h, tumor sites of mice were irradiated by 785 nm laser (1.5 W cm^{-2}) or 1064 nm laser (3.25 W cm^{-2}) for 10 min. During the course of irradiation, we utilized infrared thermal imaging cameras to monitor the temperature changes of tumor sites. We recorded the body weight and tumor volume of each mouse every 2 d during 3 weeks of treatment. The sizes of tumors were measured by a digital caliper in two dimensions, which were along the length axis (a) and orthogonal axis (b) of tumors. The tumor volumes (V) were calculated according to the following equation: $V = a*b^2/2$. Main organs (heart, liver, spleeny, lung and kidney) were harvested for H&E-stained histological evaluation after 3 weeks of treatment, which were fixed in a 4% polyoxymethylene solution and then embedded in paraffin for H&E staining. Tumor tissues were fixed with 4% paraformaldehyde in PBS and then embedded in paraffin. Tumor tissue sections were prepared on glass slides and were stained with hematoxylin and eosin. The orbit

blood of each group mice was collected and blood routine tests were performed with automatic blood cell analyzer (Mindray BC-6800, CHINA).

4T1 tumor-bearing or nude mice were purchased from Nanjing OG Pharmaceuticals, Co., Ltd. (Nanjing, China). All animal experimental procedures were conducted in conformity with institutional guidelines for the animal care and use of laboratory animals, and protocols were approved by the Institutional Ethics Committee for Laboratory Animals of Nanjing Han & Zaenker Cancer Institute (SYXK(SU)2017-0040).

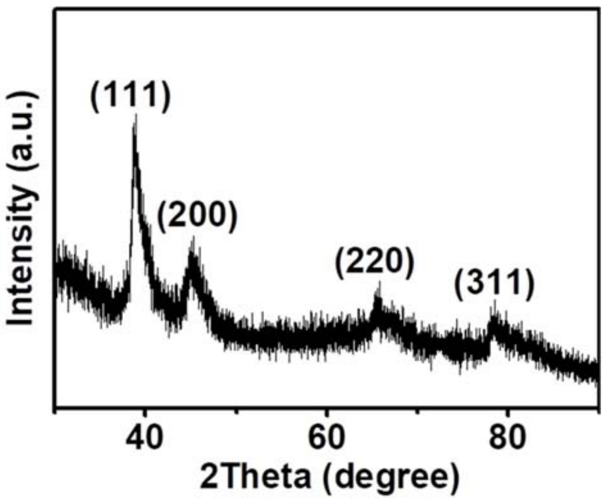

Figure S1. X-ray diffraction (XRD) pattern of PtAg nanosheets.

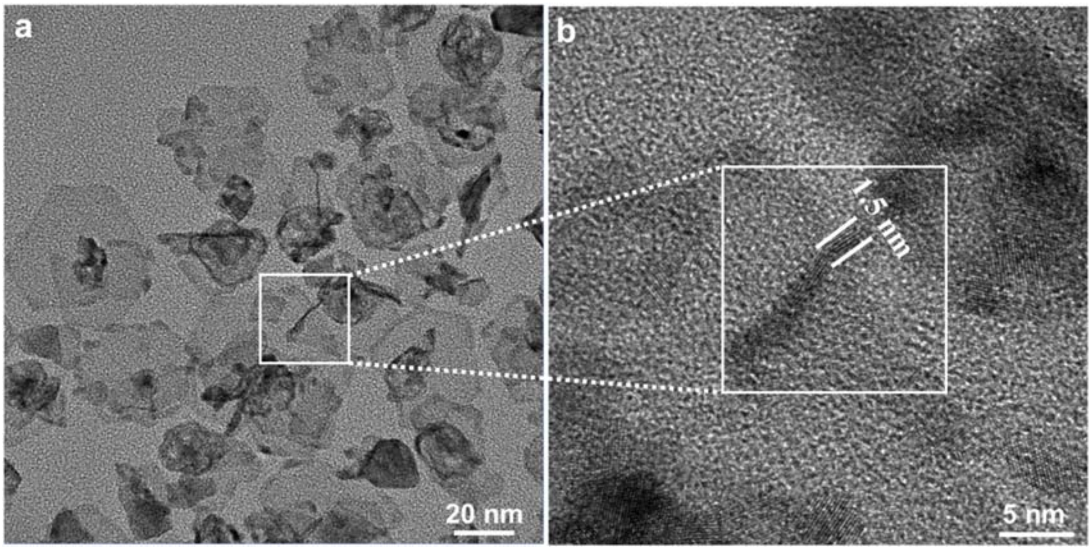

Figure S2. (a) TEM image of PtAg nanosheets. (b) Thickness analysis from HRTEM image of an erective PtAg nanosheet.

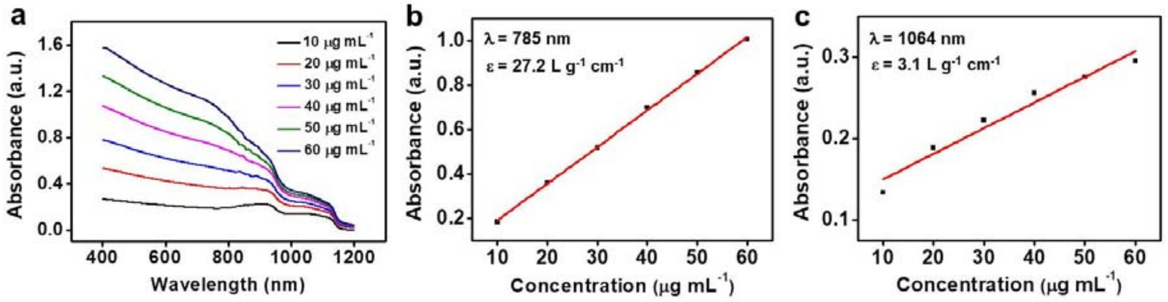

Figure S3. (a) Absorbance of PtAg nanosheet aqueous suspensions with different concentrations. The absorbance intensity at the wavelengthes of (b) 785 and (c) 1064 nm versus different concentrations of PtAg nanosheet aqueous suspensions.

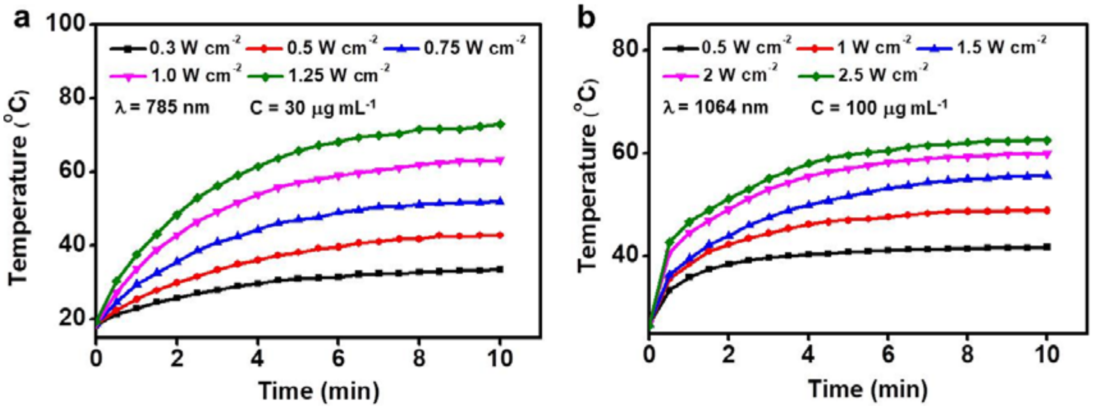

Figure S4. Photothermal heating curves of PtAg nanosheets aqueous suspensions under the irradiation of (a) 785 and (b) 1064 nm lasers with different power densities.

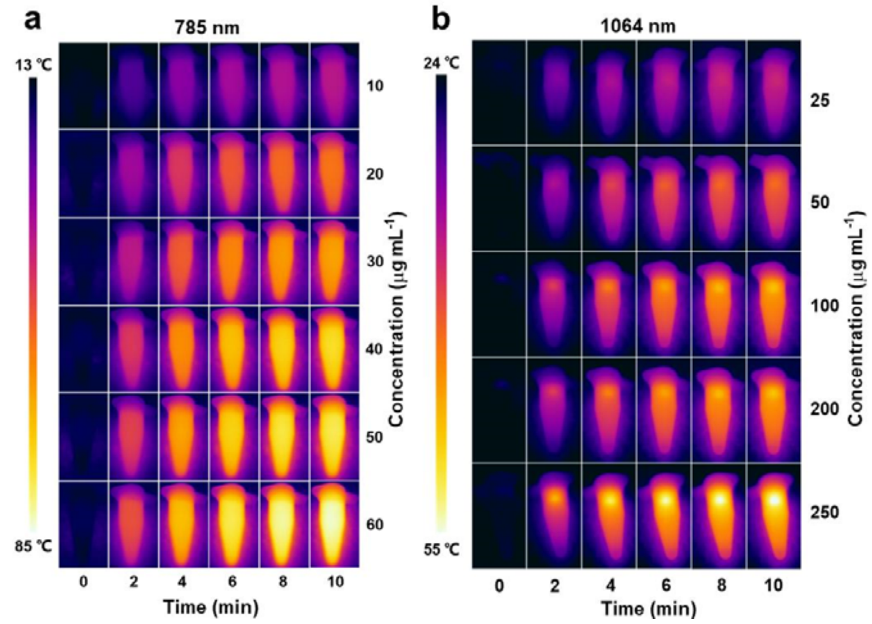

Figure S5. Photothermal images of PtAg nanosheets aqueous suspensions with different concentrations under the irradiation of (a) 785 and (b) 1064 nm lasers.

Table S1. Photothermal conversion efficiencies of PtAg nanosheets and other reported inorganic photothermal nanoagents under 1064 nm laser.

Materials	Photothermal conversion efficiency (η)	Wavelength (λ)	Reference
Pt nanoparticles with different sizes	22.98%-30.88%	1064 nm	[4]
Worm-like Pt nanoparticles	38.9%	1064 nm	[5]
Pt nanocubes	32.3%	1120 nm	[6]
Porous Pt superstructures	43.2%	1120 nm	[6]
$Au@Cu_{2-x}S$	43.25%	1064 nm	[7]
Au nanoplates@TiO_2	42.05%	1064 nm	[8]
Au-Cu_9S_5 nanoparticles	37.0%	1064 nm	[9]
Iron oxide@Au/Ag double nanoshells	28.3%	1064 nm	[10]
Cu_3BiS_3 nanorods	40.7%	1064 nm	[11]
Bi@C nanoparticles	42.32%	1064 nm	[12]
$Fe_3O_4@CuS$	19.2%	1064 nm	[13]
MoO_x nanoparticles	37.4%	1064 nm	[14]
1T-MoS_2 nanodots	43.3%	1064 nm	[15]
**PtAg nanosheets**	**45.7%**	**1064 nm**	**This work**

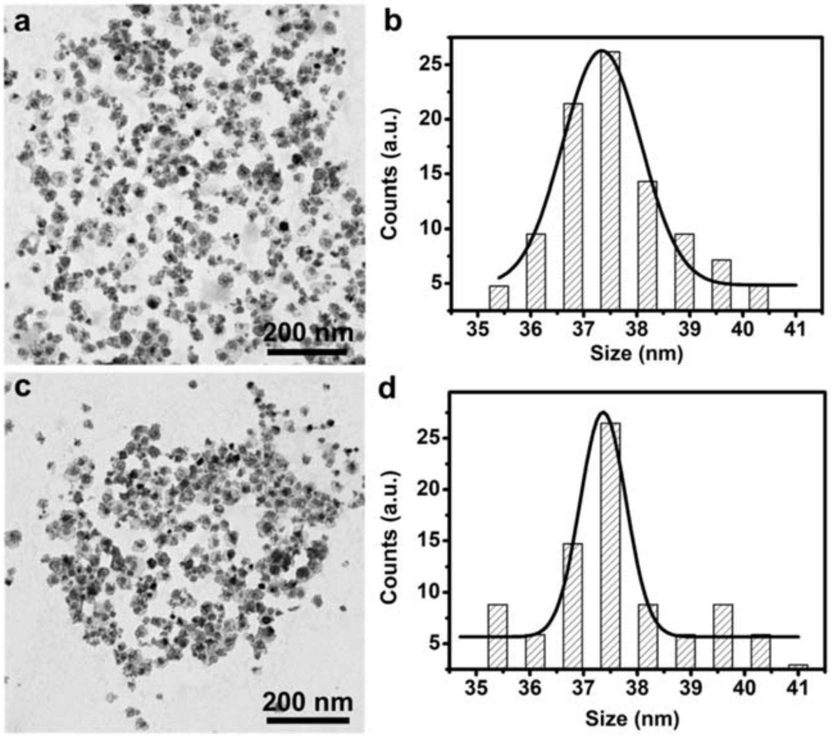

Figure S6. (a) TEM image of PtAg nanosheets and (b) their size distribution. (c) TEM image of SH-PEG-FA functionalized PtAg nanosheets and (d) their size distribution.

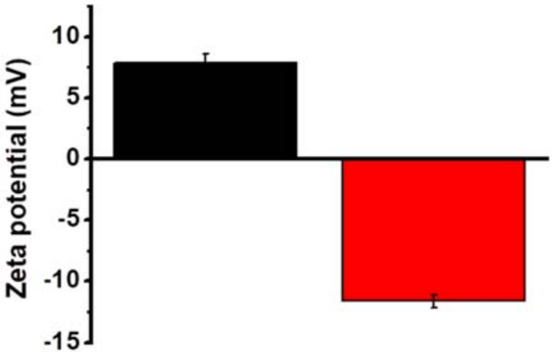

Figure S7. Zeta potential of PtAg nanosheets (black) and SH-PEG-FA functionalized PtAg nanosheets (red) in PBS (10 mM, pH = 7.4).

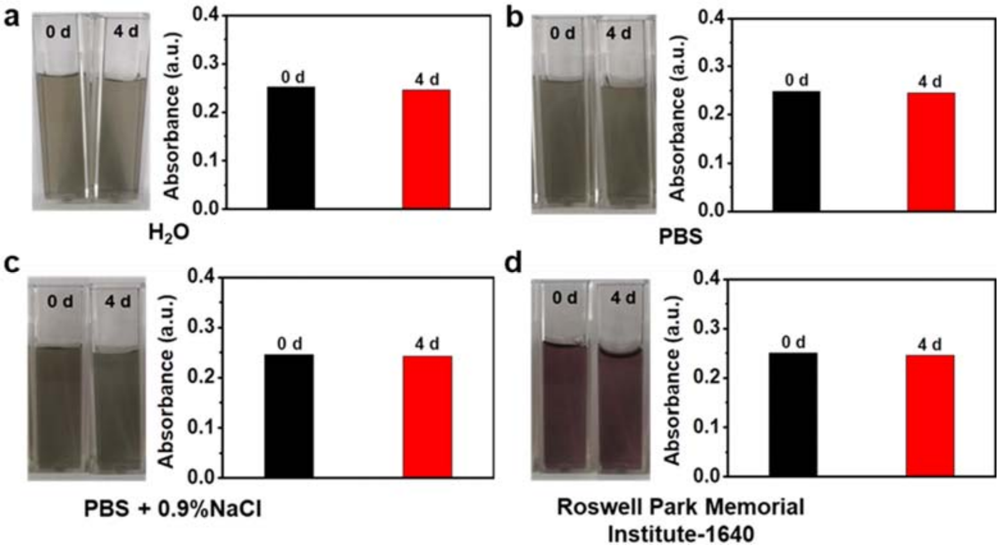

Figure S8. Photographs and absorbances at 1064 nm of SH-PEG-FA functionalized PtAg nanosheets dispersed in the (a) deionized water, (b) phosphate buffered saline (PBS) (10 mM, pH = 7.4), (c) PBS (10 mM, pH = 7.4) and physiological saline (PBS + 0.9%NaCl) and (d) Roswell Park Memorial Institute-1640 (RPMI-1640) medium for 0 and 4 d.

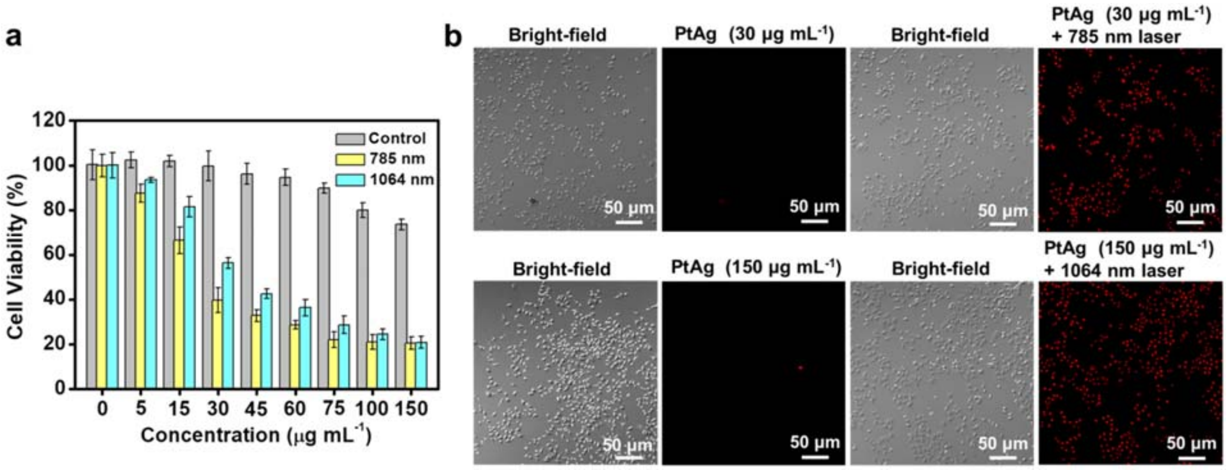

Figure S9. (a) Cell viability of HeLa cells treated with different concentrations of PtAg nanosheets under the irradiation of 785 nm laser (1.0 W cm^{-2}) or 1064 nm (1.5 W cm^{-2}) laser for 10 min. (b) Fluorescent confocal images of HeLa cells stained with PI after being incubated with and without PtAg nanosheets for 12 h, and then irradiated with 785 nm laser (1.0 W cm^{-2}) or 1064 nm laser (1.5 W cm^{-2}) for 10 min, respectively.

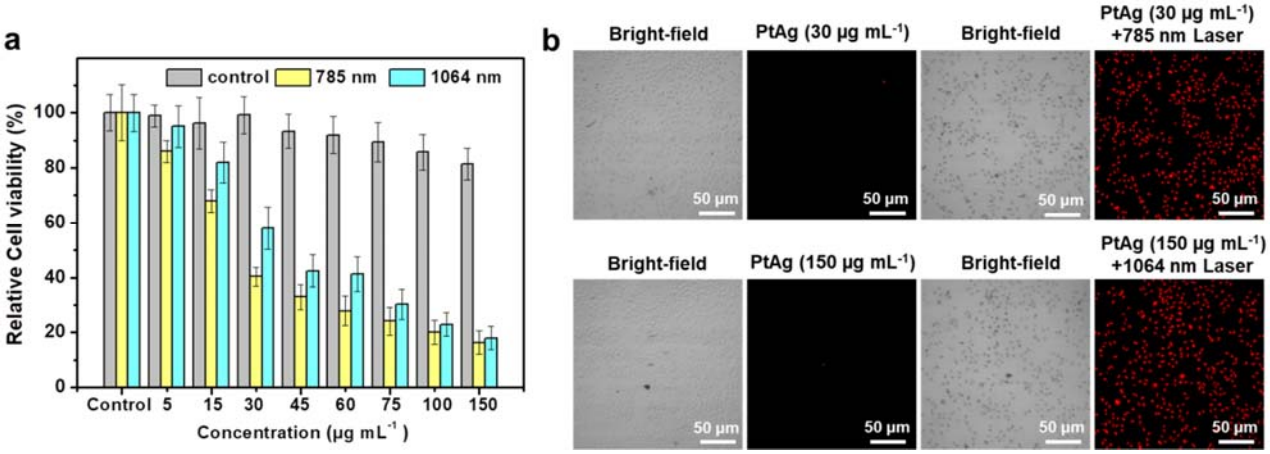

Figure S10. (a) Cell viability of 4T1 cells treated with different concentrations of PtAg nanosheets under the irradiation of 785 nm laser (1.0 W cm^{-2}) or 1064 nm laser (1.5 W cm^{-2}) for 10 min. (b) Fluorescent confocal images of 4T1 cells stained with PI after being incubated with and without PtAg nanosheets for 12 h, and then irradiated with 785 nm laser (1.0 W cm^{-2}) and 1064 nm laser (1.5 W cm^{-2}) for 10 min, respectively.

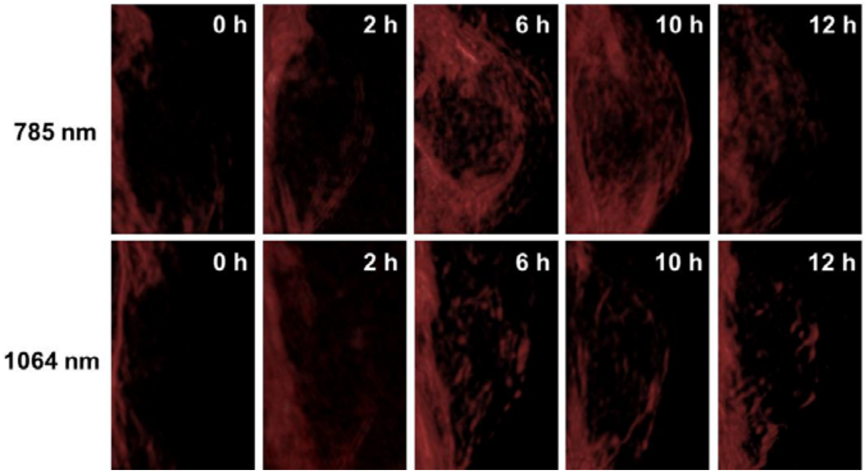

Figure S11. In vivo photoacoustic imaging of tumors under the irradiation of 785 and 1064 nm lasers after intravenous injection of PtAg nanosheets suspension for different time.

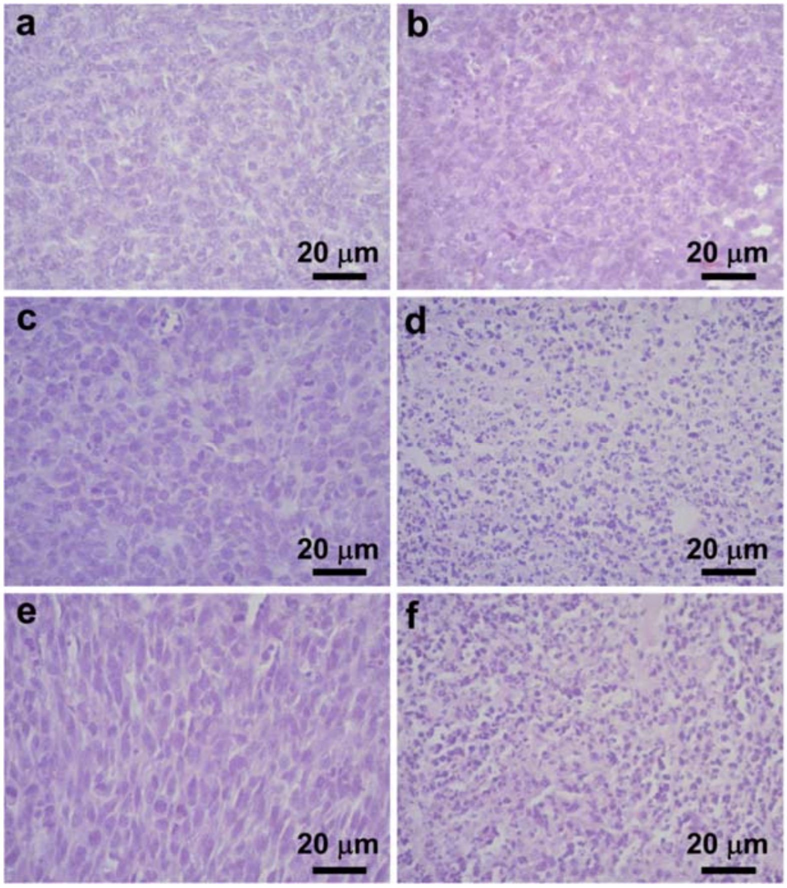

Figure S12. The hematoxylin and eosin staining of tumor tissue sections obtained from mice in different treatment groups. (a) G1: control; (b) G2: PtAg; (c) G3: 785 nm laser; (d) G4: PtAg + 785 nm laser; (e) G5: 1064 nm laser; (f) G6: PtAg + 1064 nm laser.

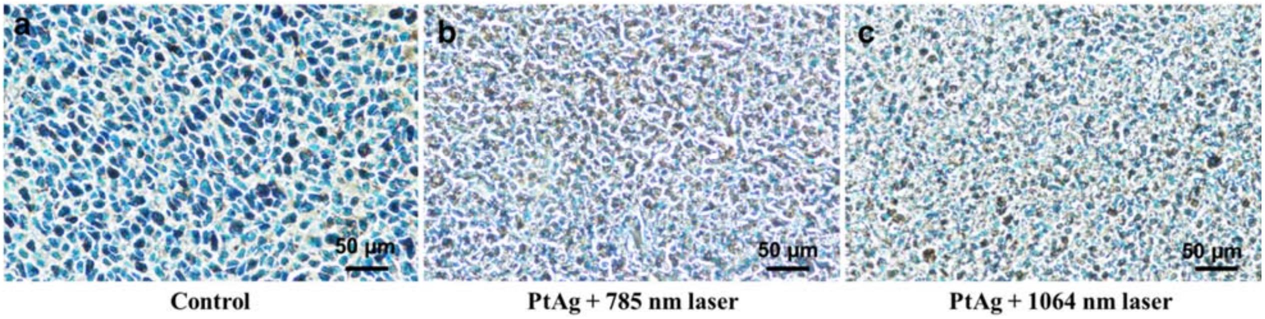

Figure S13. Proliferating cellular nuclear antigen (PCNA) assays of tumor tissue from (a) the control group and experimental group injected with SH-PEG-FA functionalized PtAg nanosheets and treated with (b) 785 or (c) 1064 nm laser.

Table S1. Analytical results of blood routines of mice with different treatments.

	G1	G2	G3	G4	G5	G6
WBC	25.53±3.94	19.97±10.24	25.27±1.62	29.33±0.45	23.9±0.56	24.87±1.19
RBC	7.74±0.24	8.34±0.1	8±0.15	6.82±0.34	7.49±0.29	7.84±0.73
HGB	131.33±2.52	139.67±4.73	134.67±2.08	126±9.54	142.67±6.51	144±9.54
HCT	0.39±0.01	0.42±0.03	0.41±0.02	0.37±0.04	0.43±0.02	0.43±0.02
MCV	51.3±1.28	51.23±0.83	52.6±0.72	51.1±0.36	52.33±0.59	51.23±0.35
MCH	17.13±0.76	16.63±0.42	16.97±0.12	16.23±0.15	16.4±0.72	16.3±0.5
MCHC	336.67±6.43	320.67±11.24	318±3.61	317.33±3.21	316.67±8.62	314.67±7.51
PLT	677±125.37	757±121.28	640±91.5	701.67±81.08	718.33±61.81	694.67±32.35
LYM%	0.12±0.01	0.14±0	0.17±0.02	0.15±0.01	0.13±0.01	0.131±0.01
NEU%	0.77±0.06	0.78±0.04	0.76±0.01	0.81±0.03	0.81±0.04	0.79±0.02
MONO%	0.05±0.02	0.05±0.01	0.06±0.01	0.06±0	0.05±0.03	0.03±0.02
EOS%	0±0	0.01±0	0±0	0±0	0±0	0±0
BASO%	0±0	0±0	0±0	0±0	0±0	0±0
LYM	2.1±0.36	2.52±1.28	3.47±0.7	4.33±0.42	3.63±0.47	3.11±0.42
NEU	19.62±1.42	19.38±2.37	17.12±4.36	21.85±2.91	17.62±5.68	20.71±2.38
MONO	0.7±0.4	0.92±0.67	1.16±0.57	1.56±0.4	0.98±0.13	0.39±0.35
EOS	0.35±0.25	0.15±0.12	0.15±0.12	0.08±0.03	0.11±0.02	0.08±0.02
BASO	0.12±0.14	0.02±0.01	0.03±0.02	0.07±0.07	0.03±0.02	0.02±0.01
RDW-SD	27.7±1.51	26.23±2.9	29.23±0.93	27.17±0.25	26.8±0.66	26.77±0.4
RDW-CV	0.16±0	0.16±0.01	0.16±0.01	0.16±0.01	0.16±0.01	0.16±0.01
PDW	14.77±0.12	15±0.3	15.13±0.25	14.9±0.1	14.93±0.32	15.03±0.21
MPV	6.27±0.21	6.33±0.32	6.6±0.44	6.73±0.25	6.33±0.21	6.7±0.2
PCT	0.43±0.11	0.39±0.15	0.4±0.06	0.38±0.04	0.38±0.05	0.44±0.05
P-LCC	42±5	182.67±233.35	44.67±6.66	50.67±4.73	42.33±3.21	42.33±0.58
P-LCR	6.67±2.08	6.33±0.58	7.33±0.58	7.33±2.08	5.33±0.58	5.67±0.58

G1: control; G2: PtAg; G3: 785 nm laser; G4: 1064 nm laser; G5: PtAg + 785 nm laser; G6: PtAg + 1064 nm laser.

References

[1] F. Saleem, Z. Zhang, X. Cui, Y. Gong, B. Chen, Z. Lai, Q. Yun, L. Gu, H. Zhang, *J. Am. Chem. Soc.* **2019**, *141*, 14496.

[2] S. Sun, L. Zhang, K. Jiang, A. Wu, H. Lin, *Chem. Mater.* **2016**, *28*, 8659.

[3] W. Ren, Y. Yan, L. Zeng, Z. Shi, A. Gong, P. Schaaf, D. Wang, J. Zhao, B. Zou, H. Yu, G. Chen, E. M. B. Brown, A. Wu, *Adv. Healthc. Mater.* **2015**, *4*, 1526.

[4] M. Manikandan, N. Hasan, H. Wu, *Biomaterials* **2013**, *34*, 5833.

[5] Q. Ma, L. Cheng, F. Gong, Z. Dong, C. Liang, M. Wang, L. Feng, Y. Li, Z. Liu, C. Li, L. He, *J. Mater. Chem. B* **2018**, *6*, 5069.

[6] Q. Wang, H. Wang, Y. Yang, L. Jin, Y. Liu, Y. Wang, X. Yan, J. Xu, R. Gao, P. Lei, J. Zhu, Y. Wang, S. Song, H. Zhang, *Adv. Mater.* **2019**, *31*, 1904836.

[7] M. Ji, M. Xu, W. Zhang, Z. Yang, L. Huang, J. Liu, Y. Zhang, L. Gu, Y. Yu, W. Hao, P. An, L. Zheng, H. Zhu, J. Zhang, *Adv. Mater.* **2016**, *28*, 3094.

[8] F. Gao, G. He, H. Yin, J. Chen, Y. Liu, C. Lan, S. Zhang, B. Yang, *Nanoscale* **2019**, *11*, 2374.

[9] X. Ding, C. Liow, M. Zhang, R. Huang, C. Li, H. Shen, M. Liu, Y. Zou, N. Gao, Z. Zhang, Y. Li, Q. Wang, S. Li, J. Jiang, *J. Am. Chem. Soc.* **2014**, *136*, 15684.

[10] M. Tsai, C. Hsu, C. Yeh, Y. Hsiao, C. Su, L. Wang, *ACS Appl. Mater. Interfaces* **2018**, *10*, 1508.

[11] A. Li, X. Li, X. Yu, W. Li, R. Zhao, X. An, D. Cui, X. Chen, W. Li, *Biomaterials* **2017**, *112*, 164.

[12] W. Y. Zhen, S. J. An, W. Wang, Y. Liu, X. D. Jia, C. Wang, M. C. Zhang, X. Jiang, *Nanoscale* **2019**, *11*, 9906.

[13] Z. C. Wu, W. P. Li, C. H. Luo, C. H. Su, C. S. Yeh, *Adv. Funct. Mater.* **2015**, *25*, 6527.

[14] W. Y. Yin, T. Bao, X. Zhang, Q. Gao, J. Yu, X. H. Dong, L. Yan, Z. J Gu, Y. L. Zhao, *Nanoscale* **2018**, *10*, 1517.

[15] Z. Zhou, B. Li, C. Shen, D. Wu, H. Fan, J. Zhao, H. Li, Z. Zeng, Z. Luo, L. Ma, C. Tan, *Small* **2020**, *16*, 2004173.
